# The Dendritic Cell-Regulatory T Lymphocyte Crosstalk Contributes to Tumor-Induced Tolerance

**DOI:** 10.1155/2011/430394

**Published:** 2011-11-03

**Authors:** Nona Janikashvili, Bernard Bonnotte, Emmanuel Katsanis, Nicolas Larmonier

**Affiliations:** ^1^Faculty of Medicine, INSERM UMR 866, IFR 100, 21000 Dijon, France; ^2^Department of Pediatrics, Steele Children's Research Center, Department of Immunobiology, BIO5 Institute, and Arizona Cancer Center, The University of Arizona, Tucson, AZ 85724-5073, USA

## Abstract

Tumor cells commonly escape from elimination by innate and adaptive immune responses using multiple strategies among which is the active suppression of effector immune cells. Regulatory T lymphocytes (Treg) and tolerogenic dendritic cells play essential roles in the establishment and persistence of cancer-induced immunosuppression. Differentiating dendritic cells (DCs) exposed to tumor-derived factors may be arrested at an immature stage becoming inept at initiating immune responses and may induce effector T-cell anergy or deletion. These tolerogenic DCs, which accumulate in patients with different types of cancers, are also involved in the generation of Treg. In turn, Treg that expand during tumor progression contribute to the immune tolerance of cancer by impeding DCs' ability to orchestrate immune responses and by directly inhibiting antitumoral T lymphocytes. Herein we review these bidirectional communications between DCs and Treg as they relate to the promotion of cancer-induced tolerance.

## 1. Introduction

Despite the arsenal harbored by the immune system to avert tumor development, cancers commonly elude immune detection and elimination by employing multiple strategies [1–5]. The past decade has witnessed considerable advances in our understanding of the mechanisms responsible for the resistance of tumor cells to immune control [6]. These include the downregulation or loss of expression by cancer cells of major histocompatibility complex (MHC) Class I molecules, resulting in the lack of recognition by cytotoxic T lymphocytes (CTL) [6–10]. Resistance to cell death (e.g., expression of antiapoptotic factors, deficiencies in the apoptosis cascade, deficiency in death receptor expression or function, blockade of perforin/granzyme) also contributes to avoidance of tumor cell killing by CTL [5, 11–15]. Additionally, cancer cells may produce immunosuppressive factors that negatively affect the function of DCs, T, and natural killer (NK) cells [11]. Nitric oxide (NO), IL-6, IL-10, tumor growth factor beta (TGF-*β*), indoleamine 2,3-dioxygensase (IDO), arginase-1, prostaglandin E2 (PGE_2_), vascular endothelial growth factor (VEGF), and cyclooxygenase-2 (COX-2) are examples of such molecules that can impede the proliferation and function of CD4^+^ and CD8^+^ T cells [5, 12, 16]. This immunosuppressive tumor environment may also foster the generation and/or promotion of immunosuppressive cells such as type 2 macrophages (M2), myeloid-derived suppressor cells (MDSCs), immature/tolerogenic DCs, and Treg [17–20]. 

By virtue of the immunosuppressive cytokines they secrete or through direct cell-cell contact interactions, both tolerogenic DCs and Treg can block antitumoral T- or NK cell activation and/or induce lymphocyte anergy or apoptosis [20–26]. Such properties place these cells at the center of tumor-induced immunosuppressive networks. Different mechanisms responsible for the accumulation of tolerogenic dendritic cells and Treg in cancer have been described but are still subjected to intensive investigation. One of them may involve a positive feedback loop by which tolerogenic DCs induce Treg that in turn contribute to the induction of immunocompromised DCs. We here review the bidirectional communications between tolerogenic DCs and Treg and their roles in the context of tumor-induced immunosuppression.

## 2. The Central Role of Regulatory T Cells and Dendritic Cells in the Induction and Maintenance of an Immunosuppressive Tumor Microenvironment

### 2.1. Tolerogenic DCs and Their Contribution to Cancer-Induced Immunosuppression

#### 2.1.1. DC Function Depends on Their Maturation and Activation Status

Known for years for their unique capability to function as professional antigen-presenting cells (APCs), DCs play a central role in the initiation and regulation of immune responses and are thereby essential for the protection against infectious pathogens and neoplastic cells [27–30]. DCs are endowed with the potential to activate antigen-specific effector T lymphocytes and are capable of promoting NKT and NK cell function [27, 31, 32]. The efficient stimulation of tumor-specific T lymphocytes by DCs requires the presentation of tumor-derived epitopes on MHC class I and II molecules together with second signals (costimulatory molecules CD80, CD86, CD40) and proinflammatory cytokines such as IL-12 or TNF-*α* [27, 31–33]. Immature DCs are characterized by high antigen uptake and processing capabilities, but by low expression of costimulatory molecules and thus are not capable of efficiently activating T cells. Multiple DC activation molecules including cytokines (such as interferons, TNF-*α*, GM-CSF, PGE_2_, or IL-1*β*), ligands of the TNF receptor family, or TLR ligands can act as “danger” signals when tissue damage occurs or pathogens are present [33–35]. These signals promote the differentiation of resident immature DCs into mature DCs characterized by the upregulation of MHC (class I and II) and costimulatory molecules (such as CD80/CD86, OX40L, ICOSL), the production of proinflammatory cytokines including IL-12, TNF-*α*, IL-1*β*, or IL-6, and the ability to migrate, in response to specific chemokines, to the secondary lymphoid organs where they encounter naïve T cells [31, 36]. Only fully matured DCs are capable of priming and activating CD4^+^ and CD8^+^ T lymphocytes [34, 37, 38]. The ability of DCs to function as inducers of immunity thus depends on their activation/maturation stage. 

Although traditionally viewed as the main inducers of immunity, DCs can also participate in the maintenance of peripheral self-tolerance [39, 40]. Under steady-state conditions, in the absence of inflammatory danger signals, immature DCs constantly engulf, process, and present self-antigens from apoptotic cells to potentially self-reactive T lymphocytes, resulting in T-cell anergy or deletion [40–42]. Migration of these immature DCs to the secondary lymphoid organs is contingent upon expression of CCR7, a chemokine receptor normally expressed by mature DCs. This mechanism is essential for the prevention of autoimmunity. In addition to anergizing antigen-specific T cells, these immature DCs have also been involved in the generation of Treg which further contributes to peripheral tolerance [43–46].

#### 2.1.2. Immature/Tolerogenic DCs in Cancer

A profound deficit in the function of DCs (lack of costimulatory molecule expression, decreased production of proinflammatory cytokines, deficiency in the antigen processing and presenting machineries, inability of activating T lymphocytes) has been described in cancer-bearing hosts [26, 47–50]. In cancer patients, tumor-derived factors have been reported to alter DC differentiation and maturation and thereby promote the accumulation of immature DCs (iDCs) in the tumor (tumor-infiltrating DCs, TiDCs) and the lymph nodes. These immunocompromised DCs are unable to initiate antitumor immune responses but can tolerize T lymphocytes [20, 26, 39, 40, 51–54] and, as discussed in Section 3, contribute to the recruitment, expansion, and function of Treg [43, 46, 55–58]. For instance, TiDCs isolated from patients with breast cancer, ovarian cancer, head and neck or lung cancer express inhibitory molecules and fail to induce autologous T-cell proliferation [51, 59, 60]. In murine tumor models a subset of immature myeloid DCs is expanded in the tumor-draining lymph nodes. These immature DCs have decreased production of IL-12, TNF-*α*, and IL-6 and increased production of IL-10 and TGF-*β* and of IDO and are responsible for the establishment of an immunosuppressive environment [61]. Upregulation of immunosuppressive molecules such as B7-H4 also contributed to the tolerogenic characteristics of these DCs [62]. Immunocompromised DCs have also been found in rat cancer models. TiDCs expressing MHC class II and ICAM-1 but lacking costimulatory molecules are not capable of inducing allogeneic T-cell proliferation [63–65]. In addition to myeloid iDCs, accumulation of plasmacytoid DCs (pDCs) has also been found in the tumor-draining lymph nodes in B16 tumor-bearing mice [66] and in head and neck human tumors [67]. These pDCs are recruited to the tumor microenvironment in response to several chemokines, including CCL20, stromal cell-derived factor-1/CXCL12, and Ag-5/vascular cell adhesion molecule-1 interactions [68, 69]. The majority of these pDCs exhibit poor immunostimulatory capacity, express IDO, and may promote FoxP3^+^ Treg rather than activating effector T lymphocytes [70, 71]. In humans, the accumulation of IDO-expressing cells in melanoma [72–74], pancreatic ductal adenocarcinoma [75], ovarian cancer [76], colon cancer [77, 78], and non-small-cell lung cancer [79] has been associated with a worsened clinical outcome. However, in contrast to these observations, IDO expression in tumor endothelial cells of patients with renal cell carcinoma seems to restrict tumor growth and to contribute to long-term survival, possibly by limiting the influx of tryptophan from the blood to the tumor or by generating metabolites toxic to tumor cells [80]. These opposite results may be explained by the type of cells expressing IDO. In fact, unlike other malignancies where the main source of IDO is either the cancer cells themselves or tumor infiltrating leukocytes (DCs, eosinophils), in renal cell carcinoma IDO is almost exclusively expressed by endothelial cells of newly formed blood vessels. IDO expression by cells involved in the microvasculature has been associated with a Th-1-related cytokine milieu (mainly IFN-*γ*) [80] which may impair tumor growth. Consistently, high microvessel density correlates with lower tumor grade and prolonged survival of patients with renal cell carcinoma [81]. Immature/tolerogenic DCs may also contribute to tumor development by fostering tumor angiogenesis. They are indeed capable of producing different cytokines and growth factors such as VEGF, promoting neoangiogenesis [82, 83].

Different approaches have been evaluated to correct the phenotypical and functional deficiencies of DCs in cancer, which include attempts to promote their maturation using different techniques. For example, the combination of CpG and anti-IL-10R antagonist has been reported to enhance IL-12 production and therefore the capacity of DCs to activate specific T cell *in vitro* and *in vivo* [84]. Interestingly, short-term ablation of DCs *in vivo* using a diphtheria toxin-based system has been reported to impair tumor growth in animal models [85].

Tumors have developed a series of strategies to suppress DC function. Some of the defined mechanisms underlying the blockade of DC maturation and the accumulation of tolerogenic DCs include the production of immunosuppressive factors such as TGF-*β*, IL-10, IL-6, VEGF, IDO, and PGE_2_ [11, 18, 70, 86]. This results in the induction of inhibitory signaling pathways in DCs. One of them involves the transcription factor STAT-3, which plays a key role in the regulation of inflammatory processes [87]. Constitutive STAT-3 activation in tumors (both of hematopoietic and of epithelial origin) inhibits the production of proinflammatory cytokines by infiltrating immune cells while promoting the release of soluble factors that suppress DC function [87–92]. Furthermore, some tumor-derived molecules (VEGF, IL-6) enhance the expression of STAT-3 in DCs [20, 91, 92]. STAT-3 activation, although an important event in early differentiation of DCs, is decreased in fully differentiated mature DCs [91]. Tumor-induced maintenance of constitutive STAT-3 activation in DCs eventually results in the acquisition of the tolerogenic potential of these cells [91, 93–98]. Expectedly, the disruption of STAT-3 signaling, for example, using dominant negative STAT-3 variants in the mouse, leads to tumor regression or growth control *in vivo * [90, 98, 99]. Similarly, the cytokine signaling inhibitor SOCS-1 has been highlighted as an important regulator of DC APC function [100]. The inhibition of this molecule using specific siRNA has been reported to break tolerance to the self-antigen Trp2 in an established B16 tumor model [100].

In addition to the mechanisms described above, tumor-induced Treg may also participate in the inhibition of DC maturation and thus in the generation of tolerogenic DCs.

### 2.2. Regulatory T Cells Critically Contribute to Tumor-Induced Tolerance

#### 2.2.1. Regulatory T Lymphocytes

Initially described in the field of autoimmunity, regulatory T cells (Treg) are comprised of a heterogeneous population of T lymphocytes defined by their capacity to suppress immune responses to self- and foreign antigens [23, 101–105]. Treg can act as critical checkpoints in the control of autoimmunity, infections, or cancer [19, 23, 101, 106–110]. A wide diversity of immunosuppressive T cells have been identified [101]. As a member of the growing family of immunosuppressive/regulatory T lymphocytes [23, 101, 107], the CD4^+^CD25^+^ Treg subset has been extensively studied over the last two decades. These cells constitute about 10% of the circulating T-lymphocyte population in mice and 5% in healthy humans [111]. In addition to CD25, the *α*-chain of the IL-2 receptor, this lymphocyte subpopulation also expresses multiple markers including cytotoxic T lymphocyte-associated antigen-4 (CTLA-4), glucocorticoid-induced TNF receptor (GITR), CD62L, lymphocyte activation gene 3 (LAG 3), Toll-like receptors (TLR-4, -5, -7, -8) [112]. In human, the IL-7 receptor (CD127) has been used to distinguish Treg from activated T cells. CD127 expression has indeed been reported to inversely correlate with FoxP3 expression and the suppressive function of Treg [113, 114]. However, increased CD127 expression has also been detected on activated (ICOS- and CD103-expressing) Treg subsets [115]. Expression of the ectonucleotidase CD39 by FoxP3^+^ Treg has been reported in mouse and human [116]. However, in contrast to mice, in human this enzyme seems to be restricted to a subset of FoxP3^+^ regulatory effector/memory-like T (Trem) cells [116]. CD39 together with another ectoenzyme (CD73) is involved in the generation of pericellular adenosine from extracellular nucleotides, resulting in the suppression of adenosine A2A receptor-expressing activated T-effector cells [117]. The forkhead/winged helix transcription factor FoxP3 appears fundamental for the development and function of CD4^+^CD25^+^ Treg and remains the most specific molecular marker for these cells [112, 118–121]. Treg contribute to the prevention of autoimmune diseases by controlling the activity of autoreactive T lymphocytes that have escaped negative selection in the thymus [103, 105, 122]. Elimination of Treg or genetic alteration of the FoxP3 gene results in the development of lethal autoimmune conditions, evidencing the essential role of these cells in the maintenance of active dominant peripheral tolerance [111, 123–125]. Depending on their origin, two types of CD4^+^CD25^+^FoxP3^+^ Treg can be identified. Naturally occurring Treg (natural or nTreg) that develop in the thymus and adaptive (inducible or iTreg) are generated by the conversion of CD4^+^CD25^−^ naïve T cells in the periphery [126–128]. It has been documented that Treg survival and immunosuppressive function and Treg production from naïve T cells depend on external signals, some of which are relayed by the TCR, CD28, TGF-*β*, and IL-2 receptors and other yet to be identified molecules [101–103, 129–132], converging towards the regulation of specific gene expression such as FoxP3. Although most iTreg are characterized by a CD25^high^ phenotype, the generation of CD25^−^ Treg by coimmunization with highly antigenic epitopes has also been reported [133]. In addition, the significance of CD25 expression by Treg is subjected to discussion, and T cells with regulatory properties have also been detected in the CD4^+^CD25^−^ subset [134–136]. The cellular and molecular bases for the suppressive activity of CD4^+^CD25^+^ Treg cells remain contentious [101, 119, 137–140]. Some proposed mechanisms include the production of inhibitory cytokines such as IL-10, TGF-*β*, and IL-35, a direct cell contact involving CTLA-4 and CD80/CD86, expression of granzymes, the depletion of IL-2 from the environment, the transfer of cAMP to the target cells, the release of nucleosides, and other yet unidentified mechanisms [23, 138, 141–148].

#### 2.2.2. Role of Treg in Cancer

Multiple studies have demonstrated that, besides their role in autoimmunity, Treg critically contribute to the immune tolerance of cancer. An increase in the number of these cells has been detected in the blood, lymph nodes, and spleen of tumor-bearing hosts and correlates with poor prognosis [24, 48, 127, 149–153]. Treg expansion observed during tumor progression may result from the proliferation of nTreg or from the conversion of CD4^+^CD25^−^FoxP3^−^ T cells into CD4^+^CD25^+^FoxP3^+^ iTreg [19, 126]. These two mechanisms may be complementary and may act in concert to achieve an optimal Treg expansion as reviewed in [102, 103, 111, 154]. In addition, it has been documented that a variety of tumors including breast cancer, melanoma, and lymphoma may recruit Treg to the tumor site. This Treg recruitment may involve a CCR4-dependent trafficking induced by CCL22 released by tumor cells and immune cells infiltrating the tumors such as macrophages and DCs [155]. This attraction of Treg by cancer cells and the modulation of Treg trafficking by tumor may be an essential element for the accumulation of Treg in the tumor microenvironment and for the mode of action of these cells in cancer [19, 106, 120, 127, 151, 156–159]. Treg impede antitumoral immune responses by suppressing the function of effectors CD4^+^, CD8^+^, and NK cells [24, 160–164] and also by inhibiting DC activation [48, 144, 165–168] as discussed in Section 4.

Since Treg represent a major obstacle for the elimination of tumors by immune cells, their therapeutic depletion or their functional inactivation using drugs or antibodies has been shown to improve responses to cancer immunotherapy including DC-based vaccines [150, 163, 169–171]. Different strategies have thus been explored to deplete/inactivate Treg *in vivo* [150, 163, 169–186]. However, the selective elimination or inactivation of Treg still constitutes a major challenge in immunotherapy since these cells share the same surface markers as activated conventional nonsuppressive T cells. Antibody-based approaches indistinctly target both Treg and activated effector T lymphocytes, and in most cases chemotherapeutic agents used to eliminate Treg do not exert specific effects on these cells. We have shown in the rat that cyclophosphamide administration results in elimination of both regulatory and effector T cells but that effector cell reconstitution occurs earlier than that of Treg [150]. Cyclophosphamide therapy enhanced tumor-specific vaccination [150]. At a low dose cyclophosphamide has been shown to trigger apoptosis of mouse Tregs *in vitro* and *in vivo* without significant changes in CD4^+^CD25^−^ cell viability [183, 187, 188]. However, clinical studies have also indicated that cyclophosphamide may not significantly affect Treg number and function [189]. Elimination of Treg based on CD25 expression results in the concurrent depletion of activated effector lymphocytes [154]. In addition, this strategy may foster tumor-driven conversion of Treg from CD4^+^CD25^−^FoxP3^−^ T cells [154, 185].

## 3. Promotion of Treg Expansion and Function by DCs

The mechanisms controlling the induction and maintenance of Treg during tumor development are still being elucidated. As outlined above, although critical for the development of adaptive immune responses, DCs may also contribute to the mechanisms of immune tolerance. These “tolerogenic” DCs of both plasmacytoid (pDCs) or myeloid (mDCs) origin are not only capable of anergizing effector T lymphocytes but may also be endowed with the capacity to drive the differentiation and/or proliferation of FoxP3^+^ Treg [39, 43, 46, 53, 58, 67, 190–199]. The ability of DCs to induce immune tolerance is believed to depend on their origin, activation state, the nature of the maturation signals and the cytokine context at the time they encounter T lymphocytes. Different subsets of tolerogenic DCs capable of promoting Treg expansion and/or function have been described [53, 57, 192, 195, 199, 200]. In physiological conditions, steady-state immature myeloid DCs constantly engulf and process self-antigens and upon migration to the draining lymph nodes can block self-reactive effector T cells and promote Treg expansion [39, 40, 58], thus contributing to the prevention of autoimmunity. In addition, semimature myeloid DCs, which exhibit some of the characteristics of mature DCs (including costimulatory molecule expression) but that produce significantly lower level of proinflammatory cytokines, have also been described for their ability to drive the differentiation of adaptive Treg [20, 39, 55, 196, 201, 202]. Importantly, phenotypically mature DCs not only induce immunity but may also exhibit a tolerogenic function. For instance, DCs isolated from Peyer's patches, lungs, or the anterior chamber of the eye display a mature phenotype, secrete IL-10, and are capable of inducing Treg [200]. CD40L-activated pDCs may also be tolerogenic and support Treg expansion [43, 203]. In addition, following extensive stimulation *in vitro* with maturation signals (e.g., LPS), DCs become “exhausted” and produce IL-10 but not IL-12 and elicit nonpolarized memory cells and/or Th2 responses [204]. Whether these “exhausted” DCs may also induce Treg *in vivo* remains however to be determined. In addition, variable results have been reported as to whether mature or immature DCs may preferentially lead to Treg induction [55, 200]. 

The mechanisms underlying DC-mediated induction of Treg are still not entirely clear. Evidence has been provided that IDO, a key-enzyme that catalyses the degradation of the essential amino acid tryptophan into kynurenine, may play an important role in this process [70, 205]. IDO-mediated tryptophan deprivation from the T-lymphocyte environment results in the downregulation of TCR-*ζ*-chain and leads to the activation of the GCN2 (general control nonrepressed 2) kinase pathway that prevents T-cell cycling and activation [206, 207]. In addition the byproducts of the tryptophan catabolism such as L-kynurenine, 3-hydroxykynurenie, or 3-hydroxyanthranilic acid may be endowed with inherent suppressive activity [206, 207]. IDO can be expressed by different DC subsets in mouse and human [208]. Although CD8^+^ DCs and plasmacytoid DCs were originally identified as the main source of IDO, it has recently been shown that CD8a^−^ IDO^−^ DCs can be converted into IDO^+^ tolerogenic DCs [209]. IDO expression has been identified as a possible factor involved in DC-mediated induction of Treg [66]. In mice and human it has been reported that IDO^+^ DCs are able to promote the differentiation of iTreg from a pool of naïve T cells [206–208, 210]. Treg induction and activation by IDO^+^ DCs require the GCN2 pathway and may be prevented by CTLA-4 blockade [66]. It has also been shown that the production of TGF-*β* by DCs conditioned by the tumor microenvironment also promotes iTreg generation [126]. TGF-*β*, together with TCR and CD28 ligation, induces an intracellular signaling that involves the cytosolic Smad proteins (Smad 2 and 3) and STAT-3 and -5 activation, resulting in FoxP3 expression [112, 118, 126, 211]. Engagement of T-cell CTLA-4 and GITR by their ligands on DCs induces the activation of preexisting Treg as well as their *de novo* generation [66, 156, 208, 210]. The engagement of programmed death receptor-1 (PD-1) expressed by T cells with B7-H1 expressed by DCs and macrophages results in the negative regulation of target T lymphocytes [212]. B7-H1-expressing DCs generated in the tumor environment exhibit reduced T-cell stimulatory capacity and have been reported to foster Treg expansion by conversion of naïve T cells into iTreg and/or by promoting the proliferation of nTreg [212–215].

The homing of Treg to the tumor site or to the tumor-draining lymph nodes where they interact with their targets is essential for their role in cancer-induced tolerance. DCs are capable of modulating the trafficking and therefore the recruitment of Treg to the tumor site or to the secondary lymphoid organs [44, 155, 216]. Blood Treg have been shown to express high CCR4 and to selectively migrate in response to the CCR4 ligand CCL22 produced by tumor cells but also by tumor infiltrating DCs [127, 217–221]. 

**Figure 1:**
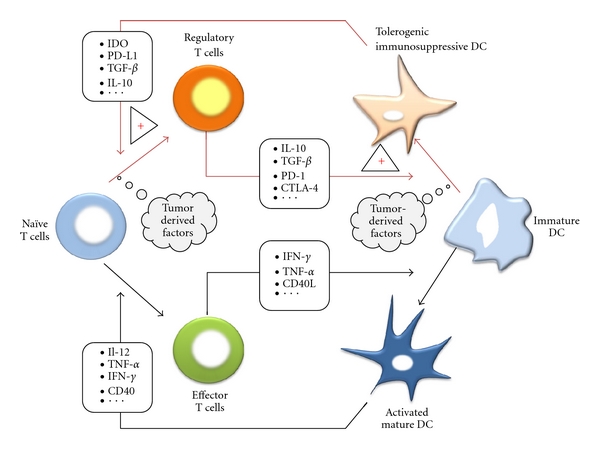
Bidirectional communications between Treg and tolerogenic DCs in cancer. Tumor-derived factors can promote the differentiation of immature DCs and naïve T cells into tolerogenic DCs and Treg. Tolerogenic DCs contribute to the generation of Treg by various mechanisms. In turn, Treg participate in tumor-induced tolerance by restraining DC maturation and fostering the accumulation of tolerogenic DCs.

In summary, DCs subverted by the tumor microenvironment lack effector T-cell stimulatory capacity but are endowed with the ability to promote suppressive Treg. In addition to tumor-derived factors which can directly induce Treg proliferation and/or generation from naïve T cells, DCs that differentiate in the tumor microenvironment provide essential signals that contribute to Treg expansion. Induction of Treg by DCs thus appears as one essential mechanism employed by cancers to generate immunosuppressive Treg and thereby to escape from antitumor immune responses (Figure 1).

## 4. Treg Negatively Modulate DC Maturation and Promote the Generation of Tolerogenic DCs

These interactions between immunosuppressive/tolerogenic DCs and Treg are not unidirectional, and Treg can “talk back” to DCs, influencing their maturation status (Figure 1). In a nontumor setting, the downregulation of DC costimulatory molecule expression [144] and IL-12 secretion [167] by Treg has been documented in the mouse. Human Treg have also been reported to exhibit suppressive effects on monocyte/macrophages [168] and on DCs generated from peripheral blood monocytes [166]. An inhibition by Treg of the maturation induced by a cocktail of TLR ligands of human myeloid but not plasmacytoid DCs has also been reported [222]. Other studies have indicated that Treg may suppress DC costimulatory molecules CD80 and CD86 without affecting CD40 expression and that inhibition of DC maturation occurs in the absence of CD40-CD40L interaction [198]. In tumor immunity, Treg have primarily been described for their ability to impair the function of tumor-specific CD4^+^ and CD8^+^ T cells [102, 106, 223]. However, it has been reported that Treg from tumor-bearing mice may impair the expression of DC costimulatory molecules CD80, CD86, and CD40, suppress DC production of proinflammatory cytokines IL-12 and TNF-*α*, and inhibit their ability to induce T-cell activation *in vitro* [48, 165]. A proposed mechanism underlying tumor-induced Treg-mediated suppression of DCs may involve the suppressive cytokines TGF-*β* and IL-10 [48]. 

Treg have also been reported to induce the expression of the immunosuppressive molecules B7-H3 and B7-H4 on DCs [44, 224–226]. B7-H3 and B7-H4 are members of the B7 family, but, in contrast to their activating counterparts, they trigger inhibitory signals in T lymphocytes and thus contribute to the immunosuppressive function of DCs and thereby to cancer-induced tolerance [44, 212, 225]. These modifications in the expression of DC surface markers may depend on diverse mechanisms, and, in addition to CTLA-4, a role for LFA-1 (lymphocyte function-associated antigen 1), LAG-3 [227], and neuropilin-1 has been proposed [227]. The engagement of the B7 molecules on DCs by CTLA-4 on Treg has been shown to upregulate IDO production in human and murine DCs which then promote Treg [206]. In turn, IDO-activated Treg have been shown to induce PD-L1 upregulation on DCs [66, 207] resulting in an efficient feedback amplification loop [66]. An additional mechanism by which Treg may promote tolerogenic DCs involves the induction of IL-10 production by DC [226]. 

Importantly, mature DCs have been shown to be refractory to Treg-mediated inhibition and seem to display a stable phenotype when exposed to these suppressive cells [144, 222]. Mouse bone-marrow-derived DCs first activated with the TLR4 ligand LPS and exposed to tumor-induced Treg maintain expression of CD80, CD86, and CD40, produce IL-12 or TNF-a, and are not impaired in their allostimulatory activity [48]. This resistance of mature DCs to Treg suppression has therapeutic implications as it underlines the importance of activating *in vitro* DCs used as vaccines prior to their administration.

Thus, Treg contribute to tumor-induced tolerance by restraining DC maturation, proinflammatory cytokine production, and APC function, therefore participating in the induction and accumulation of tolerogenic DCs.

## 5. Conclusion

There is clear evidence that DCs rendered tolerogenic by the immunosuppressive tumor microenvironment are capable not only of inhibiting effector antitumoral T cells but also of promoting the differentiation of iTreg from naïve T lymphocytes or of fostering the proliferation of nTreg. Reciprocally, cancer-induced Treg, by restraining DC maturation and by inducing DC expression and production of immunosuppressive molecules, may skew their differentiation towards a tolerogenic cell population. This positive feedback loop by which suppressed/tolerogenic DCs may induce Treg that in turn enhance DC immune inhibitory function may significantly contribute to the persistence of the immune tolerance to cancer.

These DC-Treg interactions, by enhancing tumor-induced immunosuppression, represent a major barrier to successful immunotherapy. Therefore, targeting the generation of these two suppressive cell populations is a desirable goal in chemo- and immunotherapeutic approaches. To achieve this objective there is a need to further improve strategies to simultaneously promote the full activation of DC using selective adjuvants such as TLR ligands or cytokines and impair Treg expansion, function, and recruitment.
